# Temporal trends in surfactant replacement therapy among U.S. infants, 2016–2024

**DOI:** 10.3389/fped.2026.1907867

**Published:** 2026-10-02

**Authors:** Xuezheng Sun, Wenhao Wang, Daniel Fuentes

**Affiliations:** Medical Affairs, Chiesi USA Inc, Cary, NC, United States

**Keywords:** birth certificate data, neonatal respiratory care, preterm infants, surfactant replacement therapy, temporal trends

## Abstract

**Objective:**

Surfactant replacement therapy (SRT) remains a cornerstone of neonatal respiratory management, but contemporary national data on temporal trends in SRT use across gestational age groups in the United States remain limited. We aimed to characterize gestational age–specific SRT rates among U.S. live-born infants and assess temporal trends over time.

**Study design:**

We conducted a serial cross-sectional analysis using the U.S. Centers for Disease Control and Prevention WONDER Natality Expanded database from 2016 to 2024. Findings were examined in parallel using a cohort of preterm infants from the Premier Healthcare Database from 2020 to 2024. In CDC WONDER, SRT was defined using the birth certificate item for surfactant replacement therapy. In the Premier Healthcare Database, SRT was defined as receipt of exogenous surfactant during the birth hospitalization identified from billing records. Annual percentages of infants receiving SRT were estimated by gestational age group. Temporal trends were summarized as annual percent change (APC) using Poisson regression.

**Results:**

From 2016 to 2024, the observed SRT rate increased from 0.48% (95% CI, 0.48%–0.49%) to 0.51% (95% CI, 0.50%–0.51%), an absolute difference of approximately 0.03 percentage points, corresponding to a model-estimated cumulative relative increase of 7.60% [95% CI, 6.00%–9.22%; APC, 0.92% (95% CI, 0.73%–1.11%)], with a transient deviation in 2021. The increase was most pronounced among late preterm infants born at 34–36 weeks (APC, 0.90% [95% CI, 0.45%–1.36%]) and term infants born at 37 weeks or later (APC, 3.31% [95% CI, 2.82%–3.79%]). Similar upward patterns were observed in the Premier Healthcare Database among preterm infants. The calibrated SRT rate among preterm births in 2024 was 11.39% (95% CI, 11.23%–11.54%).

**Conclusion:**

Across 2 national data sources, SRT rates increased among infants born at 34 weeks’ gestation or later. These findings suggest evolving patterns of neonatal respiratory care and inform surveillance and interpretation of national birth data.

## Introduction

Surfactant replacement therapy (SRT) is a cornerstone of neonatal respiratory care in U.S. clinical practice to treat premature infants for respiratory distress syndrome (RDS) due to insufficient surfactant production (1–3). More recently, surfactant has increasingly been used in selected late preterm and term infants with severe respiratory distress or conditions associated with secondary surfactant dysfunction such as meconium aspiration syndrome and neonatal pneumonia (4–7). This expansion has broadened both the gestational age range and the clinical contexts in which surfactant therapy is considered. In parallel, surfactant delivery has shifted toward less invasive approaches, such as thin-catheter administration while infants remain on continuous positive airway pressure (CPAP) (8–10). Taken together, these changes underscore the importance of characterizing surfactant use at the population level over time.

Population-level evidence on temporal trends in surfactant therapy use in the U.S. is limited. Prior studies have drawn on different data sources and time periods. An analysis of 538,742 infants discharged from a consortium of U.S. neonatal intensive care units (NICUs) between 1998 and 2008 reported that surfactant use increased from 16% to 19% (11). Following that, a study spanning 2008–2018 evaluated two cohorts: a clinical cohort of 259,311 infants from 359 NICUs in the Pediatrix Clinical Data Warehouse (gestational age 23 weeks 0 days to 34 weeks 6 days) and a national cohort of 1,169,441 infants with gestational age less than 35 weeks from the National Inpatient Sample (12). That study reported a decrease in surfactant therapy use from 30.8% in 2008 to 24.8% in 2018. More recently, an analysis of CDC WONDER natality data from 2016 to 2020 reported stable overall use at approximately 4.8 per 1000 live births (0.48%) (13). Notably, that study observed declining use among late-preterm infants but increasing use among term infants. Because these analyses rely on different populations and data capture settings and cover non-overlapping time periods, their findings are not comparable and do not provide a consistent, continuous picture of national trends. In addition, contemporary population-level data remains limited despite evolving neonatal respiratory care practices.

To address these gaps, we used the CDC WONDER Natality (Expanded) database to estimate annual surfactant therapy rates among all U.S. live births from 2016 to 2024, providing national context for recent utilization patterns. We also examined surfactant therapy rates among preterm births in the Premier Healthcare Database (PHD) from 2020 to 2024. Using the overlapping years between the two data sources, we assessed consistency of trends and derived calibrated national SRT rates for preterm infants.

## Materials and methods

### CDC WONDER natality database

We used National Vital Statistics System birth certificate data accessed through the CDC WONDER Natality (expanded) database for calendar years 2016 through 2024. The database is derived from the U.S. Standard Certificate of Live Birth and contains one record per live birth to U.S. residents. Gestational age (GA) was determined using the obstetric/clinical estimate from the recoded variable ‘OE Gestational Age Recode 11.’ Infants were then recategorized as: extremely preterm (<28 weeks), early preterm (28–31 weeks), moderate preterm (32–33 weeks), late preterm (34–36 weeks), or term (≥37 weeks). Surfactant therapy was identified through the checkbox item “Surfactant replacement therapy (Yes/No)” in the abnormal conditions of the newborn section of the birth certificate. Infants marked “Yes” were classified as surfactant therapy-treated (14). Infants were assigned to U.S. Census regions and divisions based on the maternal state and county of residence. Using these variables, we calculated surfactant therapy rates by year, GA groups, and census regions or divisions.

### Premier healthcare database (PHD)

The Premier Healthcare Database (PHD; Premier Inc) is a large, all-payer, hospital-based administrative and billing database that contains encounter-level information from more than 1,400 U.S. hospitals and health systems, representing approximately one quarter of all U.S. inpatient discharges and providing geographically diverse coverage (15). For this study, we included all preterm birth hospitalizations from participating hospitals contributing data in each year from 2020 to 2024. Preterm status was identified using International Classification of Diseases, Tenth Revision, Clinical Modification (ICD-10-CM) diagnosis codes P07.2x and P07.3x recorded in any diagnosis position. Infants were assigned to census divisions and regions from the hospital information, and the birth year was defined using the admission dates of hospitalizations. Accordingly, Census geography reflects maternal residence in CDC WONDER and the contributing hospital location in PHD. Gestational age was assigned based on the most specific P07.2x or P07.3x code and harmonized with the preterm GA groups same as the CDC WONDER data. The receipt of surfactant therapy was defined from hospital billing records with any billing records of exogenous surfactants, including beractant (Survanta), calfactant (Infasurf), poractant alfa (Curosurf), and lucinactant (Surfaxin).

### Statistical analysis

#### Surfactant therapy rate and trend analysis

Annual surfactant therapy rates were calculated as the number of infants receiving surfactant therapy divided by total live births in CDC WONDER and by preterm birth hospitalizations in PHD. Temporal trends in surfactant therapy rates were estimated using generalized linear models with a Poisson distribution and log link. We modeled the annual number of infants receiving surfactant therapy with an offset equal to the log of the corresponding annual denominators within each stratum, thereby estimating changes in the rate over calendar time with year modeled as a continuous predictor. Annual percent change (APC) was calculated as (exp(*β*)−1) × 100%, where *β* is the coefficient for calendar year; 95% confidence intervals were derived from model-based standard errors (16).

#### Preterm surfactant therapy rate calibration

Underreporting of birth certificate checkbox items for newborn interventions has been previously documented, with under-ascertainment particularly pronounced for infrequently reported items and for interventions occurring after transfer or later in the birth hospitalization (17, 18). During the overlapping years in CDC WONDER and PHD (2020–2024), we compared preterm surfactant therapy rates by year, Census geography, and GA group across the two data sources and calculated CDC-to-PHD rate ratios to quantify the difference.

To account for the lower ascertainment in CDC WONDER and estimate national preterm surfactant therapy rate, we fit a stratum-level binomial logistic regression model using pooled preterm strata from CDC WONDER and PHD for 2020 through 2024. Stratum was defined by data source, calendar year, GA group, and Census division. The number of infants treated by surfactant therapy was modeled as a binomial outcome with the stratum total birth as the denominator. The model included GA group, Census division, and calendar year as main effects, along with a data source indicator and its interactions with GA group, Census division, and year to allow CDC-to-PHD ascertainment differences to vary by gestational age, geography, and over time. Using the fitted model, we generated calibrated, stratum-level predicted probabilities for CDC WONDER strata. We then computed annual calibrated SRT rates for 2016–2024 overall and by gestational age group. Annual national rates were obtained as weighted averages of division-specific calibrated rates, with weights proportional to the number of births in each division. Parameter uncertainty was quantified using Monte Carlo simulation. Under an asymptotic multivariate normal approximation to the sampling distribution of the regression coefficients, we sampled 2000 coefficient vectors using the estimated coefficients and variance-covariance matrix, recalculated calibrated rates for each draw, and defined 95% confidence intervals as the 2.5th and 97.5th percentiles.

All the analysis was conducted using R version 4.5.1. This study used publicly available aggregated birth data from CDC WONDER and de-identified administrative data from the Premier Healthcare Database. All data were deidentified and adherent to the Health Insurance Portability and Accountability Act (HIPAA). Based on US Title 45 Code of Federal Regulations, Part 46, this study did not constitute human subjects research and therefore did not require institutional review board review or informed consent.

## Results

In CDC WONDER, despite a decrease in both live births and the number of infants receiving SRT from 2016 to 2024, the observed SRT rate increased from 0.48% to 0.51% (absolute difference, approximately 0.03 percentage points). The Poisson model estimated an average annual increase of 0.92% (APC, 0.92% [95% CI, 0.73%–1.11%]), corresponding to a cumulative relative increase of 7.60% (95% CI, 6.01%–9.22%) over the 9-year study period (Table 1; Figure 1; Supplementary Table S1). The surfactant therapy rate increased more consistently beginning around 2018, with a transient uptick in 2021 that may be related to the COVID-19 pandemic.

**Table 1:** Temporal trends in surfactant therapy rates by gestational age group, CDC WONDER, 2016–2024*.

Gestational age group	APC in surfactant therapy rate, % (95% CI)	Relative change in surfactant therapy rate, 2016–2024, % (95% CI)	*P* value
All births	0.92 (0.73–1.11)	7.60 (6.01–9.22)	<.001
<28 wks	1.07 (0.73–1.42)	8.91 (5.95–11.96)	<.001
28–31 wks	−0.22 (−0.59–0.15)	−1.76 (−4.64–1.21)	0.24
32–33 wks	0.24 (−0.29–0.77)	1.93 (−2.29–6.34)	0.38
34–36 wks	0.90 (0.45–1.36)	7.44 (3.62–11.39)	<.001
≥37 wks	3.31 (2.82–3.79)	29.73 (24.94–34.70)	<.001

*Surfactant therapy rate was calculated as the number of live births recorded as receiving surfactant therapy divided by total live births within each gestational age group and year. APC and relative change were estimated from Poisson regression models of surfactant counts with a log link and an offset of log(live births), with calendar year as a continuous term; relative change reflects the modeled percent change from 2016 to 2024. *P* values were from a two-sided Wald test of the calendar year coefficient (null hypothesis: APC equals 0). APC, annual percent change; CI, confidence interval; wks, weeks.

**Figure 1:**
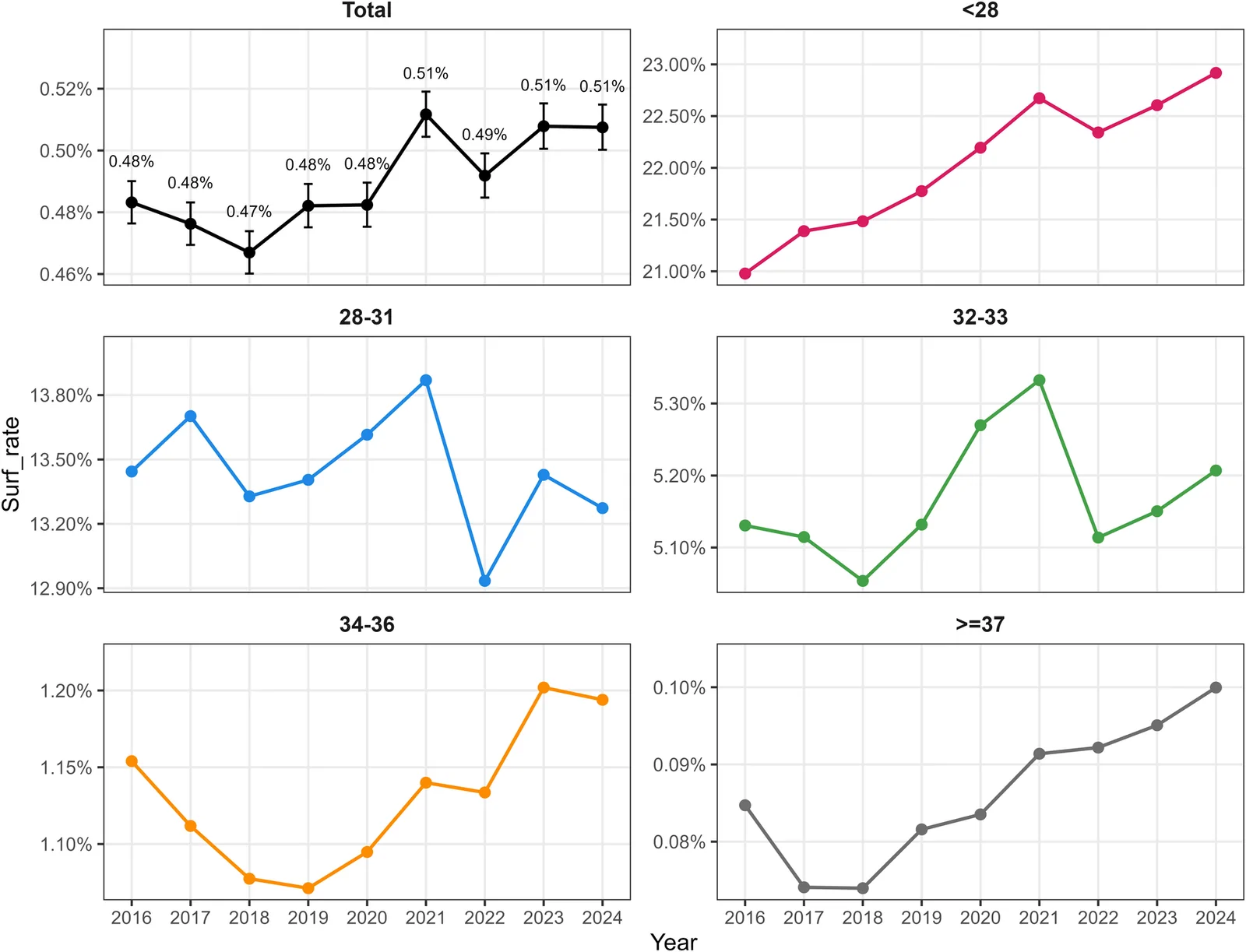
Temporal trends in surfactant therapy rate among U.S. live births by gestational age, CDC WONDER, 2016–2024. *Rates are shown as the percentage of live births recorded as receiving surfactant therapy in CDC WONDER Natality (expanded). The *x*-axis scale differs across panels. Confidence intervals for the rate were calculated using the Wilson method for binomial proportions.

In GA-stratified analyses, surfactant therapy rates increased most consistently at the extremes of gestation (Figure 1; Table 1). Among more mature infants, the APC was 3.31% (95% CI, 2.82%–3.79%) for term births (≥37 weeks) and 0.90% (95% CI, 0.45%–1.36%) for late preterm births (34–36 weeks), corresponding to cumulative relative increases of 29.73% (95% CI, 24.94%–34.70%) and 7.44% (95% CI, 3.62%–11.39%), respectively (Table 1). Rates also increased among extremely preterm infants [<28 weeks; APC, 1.07% (95% CI, 0.73%–1.42%)], although the absolute number of births and surfactant-treated infants declined (Supplementary Table S1). No significant temporal trends were observed among very preterm (28–31 weeks) or moderate preterm (32–33 weeks) infants (Table 1).

During 2020–2024, PHD included 364,724 preterm birth hospitalizations, equivalent to 19.40% of the number of preterm births recorded in CDC WONDER; 40,822 infants (11.19%) received SRT. Temporal patterns were generally similar across data sources. SRT rates increased among late preterm infants (CDC APC, 2.3% [95% CI, 0.4%–4.3%]; PHD APC, 1.6% [95% CI, 0.8%–2.4%]), whereas rates among infants born before 32 weeks were largely stable (Figure 2). To quantify potential underreporting in CDC WONDER and derive model-based calibrated estimates of preterm SRT rates (19), CDC-to-PHD ratios were calculated by year, Census region and gestational age group. CDC-to-PHD rate ratios were generally stable across years (Supplementary Tables S2, S3). After model-based calibration by gestational age, Census division, and year, the estimated 2024 preterm SRT rate was 11.39% (95% CI, 11.23%–11.54%), compared with the observed CDC WONDER rate of 4.03% (95% CI, 3.97%–4.10%) (Table 2; Supplementary Table S4).

**Figure 2:**
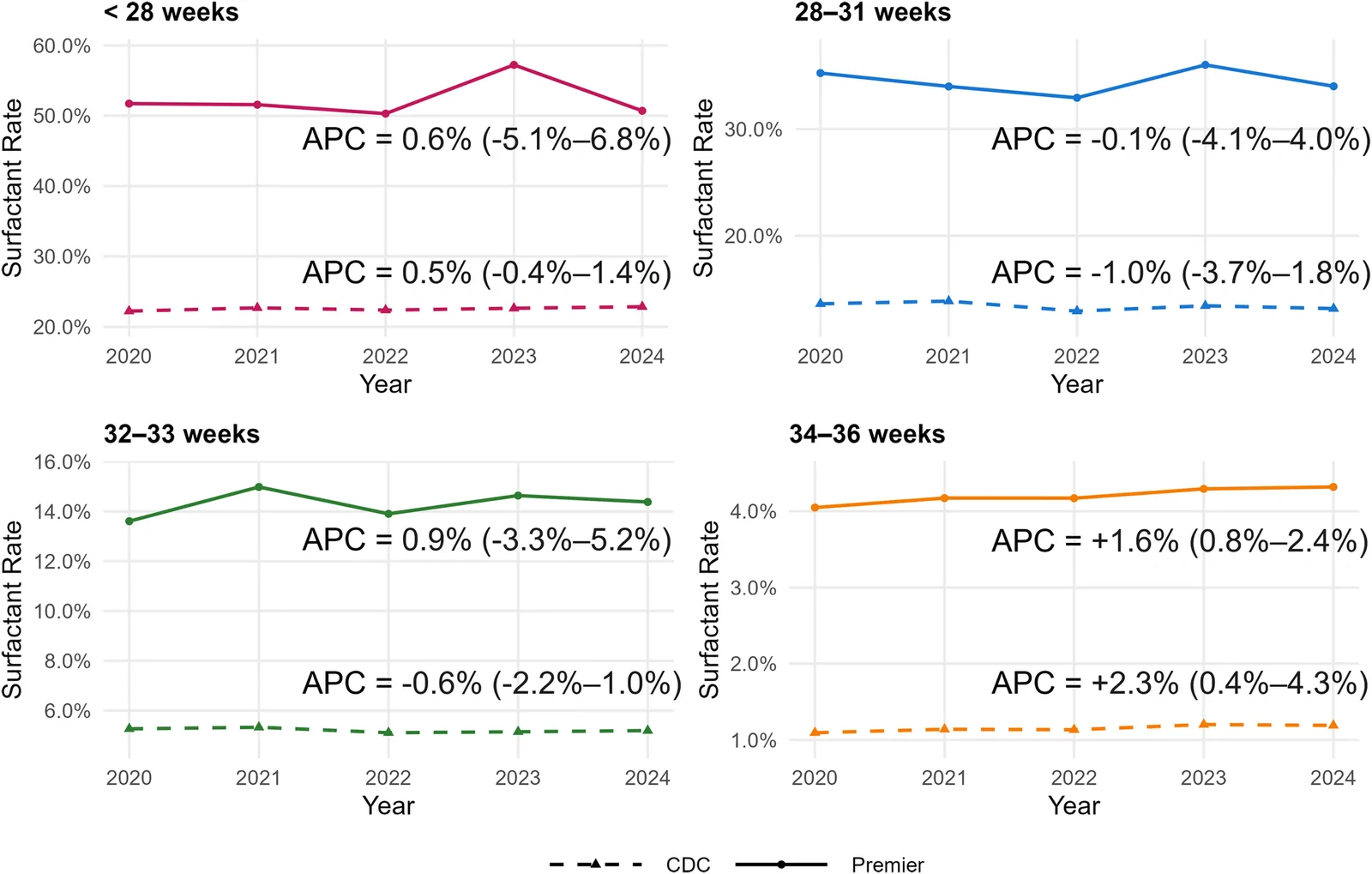
Gestational age group–specific surfactant therapy rates in CDC WONDER and the premier healthcare database, United States, 2020–2024. *Dashed lines indicate CDC WONDER natality data and solid lines indicate the Premier Healthcare Database. Rates represent the percentage of infants receiving surfactant therapy within each gestational age group and year. APC values (95% CI) denote the average annual percent change in rate during 2020–2024, estimated separately for each data source using Poisson regression with a log link and an offset for the annual denominators. APC, annual percent change; CI, confidence interval.

**Table 2:** Observed and calibrated national surfactant therapy rates among preterm infants, United States, 2016–2024*.

Year	CDC Observed preterm surfactant therapy rate, % (95% CI)	Calibrated preterm surfactant therapy rate, % (95% CI)
2016	4.15 (4.09–4.21)	10.59 (10.23–10.99)
2017	4.14 (4.08–4.21)	10.69 (10.39–11.03)
2018	4.01 (3.95–4.08)	10.73 (10.49–11.01)
2019	4.01 (3.95–4.08)	10.84 (10.65–11.05)
2020	4.05 (3.99–4.12)	10.92 (10.77–11.07)
2021	4.12 (4.05–4.18)	11.06 (10.95–11.17)
2022	3.96 (3.90–4.02)	11.16 (11.08–11.26)
2023	4.08 (4.02–4.14)	11.37 (11.26–11.48)
2024	4.03 (3.97–4.10)	11.39 (11.23–11.54)

*CDC-observed SRT rates were calculated as the number of infants recorded as receiving SRT divided by preterm live births in each year; 95% CIs were calculated using the Wilson score method. Calibrated rates were estimated from the calibration model, with 95% CIs derived using Monte Carlo simulation. Rates are calculated among all preterm live births, including the large late-preterm group with relatively low SRT use; the calibrated column accounts for lower SRT ascertainment in birth-certificate data.

## Discussion

In this national, population-based study using CDC WONDER natality data, we estimated gestational age-specific surfactant therapy rates among U.S. live births and described temporal trends from 2016 to 2024. Overall surfactant use increased modestly, with more pronounced increases among late preterm and term infants. These patterns were corroborated in a parallel analysis of the PHD from 2020 to 2024. By combining a national birth registry with a large hospital-based database, our study provides complementary evidence of how surfactant utilization is evolving across gestational ages in contemporary U.S. practice.

Most prior analyses on surfactant therapy have relied on NICU-based clinical networks. Because NICU-based cohorts disproportionately include younger preterm infants with more severe respiratory illness, reported surfactant therapy rates in these studies are substantially higher and do not reflect use in the overall infant population (20, 21). This limitation is particularly relevant for more mature infants and those cared for in lower-acuity or less-resourced settings, who are more likely to be treated in delivery rooms, newborn nurseries, or community hospitals (22, 23). Vidavalur examined the CDC WONDER natality database covering all live births from 2016 to 2020. (13) Our analysis extended the observation window by examining data from 2016 to 2024. Overall surfactant use in that study was approximately 0.48% during 2016–2020, consistent with our estimates over the same period. In contrast to the previously reported decline among late-preterm infants, rates in this group rebounded and increased steadily after 2019. The increase observed among term infants persisted beyond 2020 and reached 999.6 per 100,000 infants in 2024. We additionally observed a continued increase in surfactant therapy use among extremely preterm infants. These updated national estimates provide a more current picture of surfactant utilization amid evolving neonatal respiratory care and help inform population-level monitoring and healthcare resource optimization.

Several factors may contribute to the gestational age-specific increases in surfactant therapy, particularly among late preterm and term infants. One likely contributor is a shifting maternal and obstetric risk profile that increases neonatal respiratory morbidity and the need for escalation of respiratory support. The prevalence of maternal cardiometabolic conditions, including gestational diabetes, pre-pregnancy obesity, and pregnancy-associated hypertension, has increased and is associated with neonatal respiratory morbidity (24–29). In addition, older maternal age and greater use of assisted reproductive technology are associated with higher rates of obstetric intervention, including cesarean delivery, which is a recognized risk factor for respiratory morbidity among late preterm and early term infants (30–32). Moreover, rising surfactant therapy rates in more mature infants likely reflects evolving treatment thresholds related to secondary surfactant dysfunction. In conditions such as meconium aspiration syndrome and neonatal pneumonia, aspirated material and inflammatory alveolar exudates can inactivate endogenous surfactant, increase alveolar surface tension, and promote atelectasis, providing a physiologic rationale for exogenous surfactant therapy (33, 34). The expanding adoption of noninvasive respiratory support and minimally invasive surfactant administration, which avoids routine endotracheal intubation, may further lower thresholds for surfactant treatment. Consistent with this shift, a recent U.S. survey of neonatology providers reported that 39% used minimally invasive surfactant therapy in 2023–2024, compared with 15% in 2016 (35, 36). Together, these trends suggest that increasing surfactant use among late preterm and term infants reflects both changing clinical risk profiles and evolving respiratory management practices. Further research is needed to clarify the relative contributions of these factors and their implications for neonatal outcomes and healthcare utilization.

Prior evaluations of the revised U.S. Standard Certificate of Live Birth have shown that medical and newborn items are variably and incompletely recorded, particularly for interventions that occur after delivery room care or during the birth hospitalization (37). By pairing the CDC WONDER natality data with a large hospital-based administrative database that more directly captures billed surfactant therapy, we confirmed our findings, and placed national trends on a clinically interpretable scale while accounting for nonuniform ascertainment across gestational ages and geographic regions. By applying GA- and region-specific calibration, we derived national preterm surfactant therapy estimates on a clinical scale. In 2024, the calibrated preterm surfactant therapy rate was 11.39% (95% CI, 11.23%–11.54%), substantially higher than the corresponding CDC observed estimate among preterm births of 4.03% (95% CI, 3.97%–4.10%), and more consistent with rates reported in prior hospital-based cohorts (11, 12). Despite the value of calibrated rates in providing a model-based population-level benchmark for population-level surveillance and healthcare planning, these estimates should be interpreted in light of limitations. We evaluated variation across regions, GA groups, and year, and relied calibration on the assumption that ascertainments between two data sources are adequately captured by the model and can be transported to the national population. Moreover, calibrated estimates may still be affected by residual uncertainty due to unmeasured differences in hospital profile and care settings that were not captured in the calibration approach. Lastly, the code-based analyses of PHD may be subject to potential biases due to coding errors, inconsistency, or incomplete clinical details.

## Conclusions

Surfactant therapy use in the U.S. continues to evolve, with increasing utilization extending into more mature gestational age groups, highlighting shifts in neonatal respiratory management. Contemporary national estimates provide essential context for evaluating practice patterns and informing research on outcomes and resource utilization. Future studies are needed to characterize indications and care settings for surfactant use, clarify clinical drivers of these trends, and assess associated neonatal outcomes.

## Data Availability

The data analyzed in this study is subject to the following licenses/restrictions: the CDC WONDER Natality (Expanded) data used in this study are publicly available from the CDC WONDER online interface. Data from the Premier Healthcare Database are proprietary and were accessed under a data use agreement with Premier Inc; restrictions apply to their availability, and they cannot be shared publicly. Analytic code and aggregate results sufficient to reproduce the main findings are available from the corresponding author on reasonable request, subject to applicable data use and confidentiality restrictions. Requests to access these datasets should be directed to Premier Inc; solutioncenter@premierinc.com.
