# Supplementary material for: Temporal trends in surfactant replacement therapy among U.S. infants, 2016–2024
Source: Front Pediatr. 2026 Oct 2;14:1907867. doi: 10.3389/fped.2026.1907867 (PMC13672566; doi:10.3389/fped.2026.1907867)
Supplement: Supplementary file 1 [file Table1.docx]

**Supplementary Material**

**Temporal Trends in Surfactant Replacement Therapy among U.S. Infants, 2016–2024**

Xuezheng Sun^1^, Wenhao Wang^1^, Daniel Fuentes^1^

^1^Chiesi USA Inc, Cary, North Carolina

**eTable 1.** Temporal trends in the annual number of infants receiving surfactant therapy by gestational age group, CDC WONDER, 2016–2024

| **Gestational age group** | **APC in number of infants receiving surfactant therapy,**  **% (95% CI)** | **Relative change in number of infants receiving surfactant therapy, 2016–2024,**  **% (95% CI)** | **P value** |
| --- | --- | --- | --- |
| All births | −0.14 (−0.33 to 0.05) | −1.11 (−2.58 to 0.38) | 0.14 |
| <28 wks | −0.86 (−1.20 to −0.51) | −6.65 (−9.20 to −4.03) | <.001 |
| 28–31 wks | −1.28 (−1.65 to −0.91) | −9.78 (−12.44 to −7.05) | <.001 |
| 32–33 wks | −0.47 (−0.99 to 0.06) | −3.69 (−7.69 to 0.49) | 0.08 |
| 34–36 wks | 0.91 (0.45 to 1.37) | 7.50 (3.68 to 11.46) | <.001 |
| ≥37 wks | 2.15 (1.67 to 2.64) | 18.58 (14.18 to 23.14) | <.001 |

**eTable 2.** CDC-to-PHD surfactant therapy rate ratios among preterm infants by gestational age group and U.S. Census region, 2020–2024

| **GA Group** | **All Regions** | **Midwest** | **Northeast** | **South** | **West** |
| --- | --- | --- | --- | --- | --- |
| <28 wks | 0.45 | 0.63 | 0.68 | 0.34 | 0.43 |
| 28-31wks | 0.39 | 0.49 | 0.69 | 0.29 | 0.36 |
| 32-33 wks | 0.36 | 0.48 | 0.59 | 0.26 | 0.44 |
| 34-36 wks | 0.27 | 0.35 | 0.49 | 0.19 | 0.35 |
| All preterm | 0.37 | 0.49 | 0.61 | 0.27 | 0.40 |

**eTable 3.** Gestational age–specific surfactant therapy rates in CDC WONDER and the Premier Healthcare Database (PHD), with CDC-to-PHD rate ratios, 2020–2024.

| **Year** | **Surfactant therapy**  **rate in CDC** | **Surfactant therapy**  **rate in PHD** | **CDC-to-PHD ratio** |
| --- | --- | --- | --- |
| **Extremely preterm (<28 weeks)** | | | |
| 2020 | 23.61% | 54.43% | 0.43 |
| 2021 | 23.95% | 56.00% | 0.43 |
| 2022 | 23.65% | 55.74% | 0.42 |
| 2023 | 23.91% | 63.15% | 0.38 |
| 2024 | 24.32% | 57.55% | 0.42 |
| **Very preterm (28-31 weeks)** | | | |
| 2020 | 13.62% | 36.42% | 0.37 |
| 2021 | 13.88% | 36.96% | 0.38 |
| 2022 | 12.95% | 35.73% | 0.36 |
| 2023 | 13.44% | 39.25% | 0.34 |
| 2024 | 13.29% | 37.57% | 0.35 |
| **Moderate preterm (32-33 weeks)** | | | |
| 2020 | 5.27% | 13.62% | 0.39 |
| 2021 | 5.34% | 14.88% | 0.36 |
| 2022 | 5.12% | 14.48% | 0.35 |
| 2023 | 5.15% | 14.72% | 0.35 |
| 2024 | 5.21% | 14.61% | 0.36 |
| **Late preterm (34-36 weeks)** | | | |
| 2020 | 1.10% | 3.97% | 0.28 |
| 2021 | 1.14% | 4.01% | 0.28 |
| 2022 | 1.14% | 3.99% | 0.28 |
| 2023 | 1.20% | 4.17% | 0.29 |
| 2024 | 1.19% | 4.23% | 0.28 |
| **All preterm** | | | |
| 2020 | 4.05% | 11.96% | 0.34 |
| 2021 | 4.12% | 12.06% | 0.34 |
| 2022 | 3.96% | 11.70% | 0.34 |
| 2023 | 4.08% | 12.56% | 0.32 |
| 2024 | 4.03% | 11.16% | 0.36 |

**eTable 4.** Calibrated national surfactant therapy rates among preterm infants by gestational age group, 2024.

| **GA group (weeks)** | **No. of births** | **Calibrated No. of infants receiving surfactant therapy (95% CI)** | | **Calibrated surfactant therapy rate, % (95% CI)** |
| --- | --- | --- | --- | --- |
| <28 | 21,456 | 12,888 (12,633–13,145) | 60.07 (58.88–61.27) | |
| 28–31 | 32,858 | 12,281 (11,979–12,579) | 37.38 (36.46–38.28) | |
| 32–33 | 42,933 | 6,200 (5,959–6,444) | 14.44 (13.88–15.01) | |
| 34–36 | 278,336 | 11,405 (11,037–11,795) | 4.10 (3.97–4.24) | |
| All preterm | 375,583 | 42,774 (42,181–43,344) | 11.39 (11.23–11.54) | |
